# Maxillary Cystic Ameloblastic Fibroma in an 8-Year-Old Girl: A Case Report Featuring a Rare Histological Variant

**DOI:** 10.1155/crid/7645367

**Published:** 2025-07-08

**Authors:** Nasser Raqe Alqhtani

**Affiliations:** Department of Oral and Maxillofacial Surgery and Diagnostic Sciences, College of Dentistry, Prince Sattam Bin Abdulaziz University, Al-Kharj, Saudi Arabia

**Keywords:** ameloblastoma, dentigerous cyst, fibroma

## Abstract

**Objective:** This report is aimed at outlining the unusual cystic variant of ameloblastic fibroma to facilitate its demarcation from other odontogenic lesions, including dentigerous cysts and cystic ameloblastomas.

**Case Report:** An 8-year-old girl with no significant medical history presented to the oral surgery department with a painless swelling in the right maxillary region, first noticed 1 month ago, which gradually increased in size, accompanied by monocortical expansion of the buccal cortex. Cone beam computed tomography revealed a well-demarcated unilocular low-density lesion in the right posterior maxilla, measuring approximately 3 × 2 cm; the central bony lesion involved an unerupted first permanent molar. Conservative enucleation of the lesion was performed, along with the removal of the impacted tooth. Microscopic examination showed a benign mixed cystic odontogenic tumor, displaying odontogenic epithelial strands with stellate-shaped fibroblasts in a myxoid cell-rich stroma. The epithelial cells were rounded to cuboidal, with no mitotic activity or signs of malignancy. The overall histological image suggested a cystic ameloblastic fibroma.

**Conclusion:** Clinically and radiographically, cystic ameloblastic fibroma may resemble a dentigerous cyst due to the involvement of an impacted tooth with the lesion. However, these two entities can be clearly histologically differentiated, as the distinctive odontogenic epithelial strands in a myxoid cell-rich stroma that are seen in cystic AF will be absent in a dentigerous cyst.

## 1. Introduction

Ameloblastic fibroma (AF) is an uncommon mixed benign odontogenic tumor formed by neoplastic odontogenic epithelium and mesenchyme, excluding dental tissues such as enamel or dentine [1, 2]. It is a painless, slowly growing tumor that is less aggressive than ameloblastoma, primarily detected in the first decade of life with a slight male predilection causing retardation of tooth eruption. It can occur in both jaws, with the mandibular posterior region being the most frequent site [3, 4].

Radiographically, AF resembles ameloblastoma and dentigerous cysts, which have well-defined radiographic borders and are associated with an impacted tooth. The treatment of choice for this lesion is conservative excision. Recurrence is uncommon (0%–18%), and there may be a potential for malignant transformation [5–7].

The purpose of this report is to outline the unusual cystic variant of AF to distinguish it from other odontogenic lesions, including dentigerous cysts and cystic ameloblastomas.

## 2. Case Report

**Figure 1:**
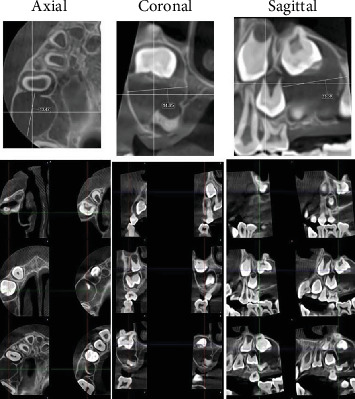
Axial, coronal, and sagittal CBCT images showing a well-defined unilocular radiolucency in the right posterior maxilla associated with the unerupted upper right first molar.

An 8-year-old girl with no significant medical history presented to the oral surgery department with a painless swelling in the right maxilla, which had been noticed 1 month ago and had gradually increased in size, without any history of tooth extraction or trauma. The proposed operation or investigations were explained in simple language which could be understood by the patient's relatives, and informed consent was obtained from the patient's parents. Upon extraoral examination, a localized firm swelling in the right posterior maxilla revealed mild facial asymmetry. Intraorally, a painless swelling that was firm to hard, exhibiting monocortical expansion of the buccal cortex, was identified in the right posterior maxilla. No signs of infection or inflammation were present, and the overlying mucosa appeared normal in color and texture. Radiographically, axial and coronal cone beam computed tomography (CBCT) showed a well-demarcated unilocular low-density lesion in the right posterior maxilla, measuring approximately 3 × 2 cm. The central bony lesion involves an unerupted first permanent molar with less than one-third of the root formed (Figure 1).

**Figure 2:**
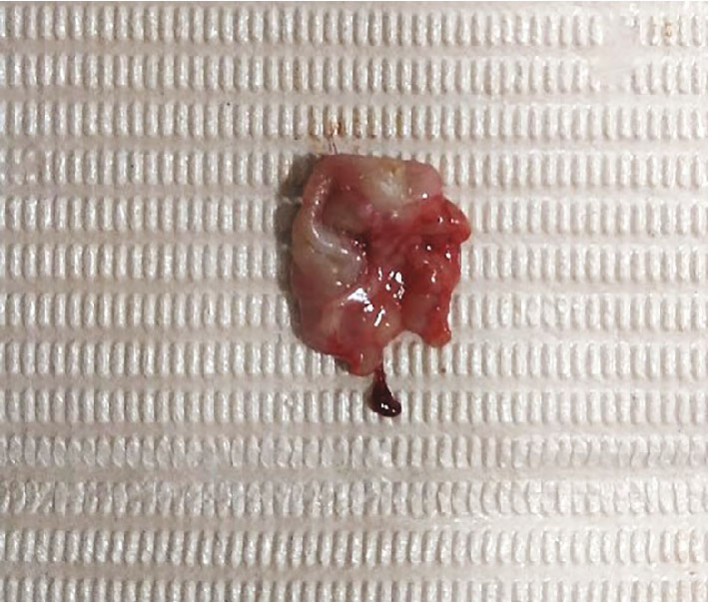
Clinical photo of the excised mass.

Conservative enucleation of the lesion was performed under aseptic precautions using a local anesthetic solution containing lidocaine hydrochloride 2% and epinephrine 1:100,000. A full-thickness buccal mucoperiosteal flap was reflected, and the alveolar bone was removed. Careful enucleation of the lesion, along with the removal of the unerupted upper right first molar, was conducted, followed by primary wound closure. The excised mass was immediately fixed in a 10% formalin solution for histopathological examination (Figure 2).

**Figure 3:**
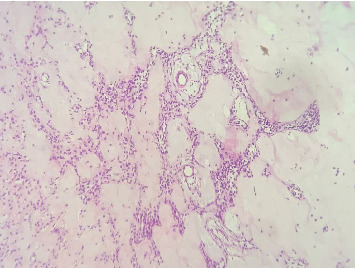
Histopathologic appearance of the biopsy depicting an odontogenic epithelial strand within a myxoid cell-rich stroma featuring stellate-shaped fibroblasts in fibrous tissue (H&E; original magnification ×20).

Microscopic examination revealed a benign mixed cystic odontogenic tumor exhibiting odontogenic epithelial strands with stellate-shaped fibroblasts within a myxoid cell-rich stroma. The epithelial cells were rounded to cuboidal, demonstrating neither mitotic activity nor signs of malignancy. The overall histological appearance indicated a cystic AF (Figure 3).

## 3. Discussion

The characteristic histopathological pattern of AF, which comprises both neoplastic epithelial and ectomesenchymal components, features cuboidal to columnar odontogenic epithelial strands in a myxoid cell-rich stroma with stellate-shaped fibroblasts in a myxoid stroma that resembles the developing dental papilla. It is regarded as less aggressive than ameloblastoma and enlarges through gradual expansion with asymptomatic clinical progression, with the patient's initial complaint being pain or swelling [5, 8].

Several unusual histological variants of AF reported in the literature include a “papilliferous AF,” as termed by Christ et al. [9]. This variant comprises papillary projections formed by the odontogenic epithelium into the cyst lumen. The cystic AF, first named by Chen et al. [10], illustrates a dentigerous cyst characterized by intraluminal growth originating from the cyst wall. The histopathological features of our case align with those described for cystic AF, with intraluminal growth comprising subtle fibrils and stellate connective tissue resembling dental papilla, texturized by ameloblast-like odontogenic epithelium.

Cyst formation in AF is uncommon; it may have been initiated in a pre-existing odontogenic dentigerous cyst lining or due to a degenerative process. However, the absence of an epithelial lining, the diffuse nature of the neoplasm, and the presence of extensive hyalinization support a degenerative process [7, 11, 12]. Usubütün et al. in 2002 [7] characterized cystic AF that involves a pseudocyst with odontogenic epithelium akin to AF. In 2022, Ali et al. [4] reported that cystic AF may present in two subtypes: The first is intraluminal cystic ameloblastic fibroma (ICAF), in which the AF components are seen in the cyst wall, and the second is mural cystic ameloblastic fibroma (MCAF), where the AF components appear in both the cyst wall and the cyst lining.

## 4. Conclusion

This case report illustrates a cystic AF in an 8-year-old girl. This aligns with the belief that AF frequently occurs in children and young adults, with no gender predilection. Clinically and radiographically, the lesion may resemble a dentigerous cyst due to the involvement of an impacted tooth with the lesion. Histologically, these two entities can be clearly differentiated, as the distinctive odontogenic epithelial strands in a myxoid cell-rich stroma seen in cystic AF will be absent in a dentigerous cyst. Therefore, careful microscopic examination of biopsied tissue is of utmost importance for establishing a definitive diagnosis.

## Data Availability

The data that support the findings of this study are available from the corresponding author upon reasonable request.
